# Developmental independence of median fins from the larval fin fold revises their evolutionary origin

**DOI:** 10.1038/s41598-022-11180-1

**Published:** 2022-05-07

**Authors:** Kazuhide Miyamoto, Koichi Kawakami, Koji Tamura, Gembu Abe

**Affiliations:** 1 Laboratory of Organ Morphogenesis, Department of Ecological Developmental Adaptability Life Sciences, Graduate School of Life Sciences, Tohoku University, Aobayama Aoba-ku, Sendai, 980-8578 Japan; 2 Laboratory of Molecular and Developmental Biology, National Institute of Genetics, Mishima, Shizuoka 411-8540 Japan; 3 Department of Genetics, The Graduate University for Advanced Studies, SOKENDAI, Mishima, Shizuoka 411-8540 Japan; 4 Present Address: Division of Developmental Biology, Department of Functional Morphology, School of Life Science, Faculty of Medicine, Tottori University, Nishi-cho 86, Yonago, 683-8503 Japan

**Keywords:** Evolutionary developmental biology, Limb development

## Abstract

The median fins of modern fish that show discrete forms (dorsal, anal, and caudal fins) are derived from a continuous fold-like structure, both in ontogeny and phylogeny. The median fin fold (MFF) hypothesis assumes that the median fins evolved by reducing some positions in the continuous fin fold of basal chordates, based on the classical morphological observation of developmental reduction in the larval fin folds of living fish. However, the developmental processes of median fins are still unclear at the cellular and molecular levels. Here, we describe the transition from the larval fin fold into the median fins in zebrafish at the cellular and molecular developmental level. We demonstrate that reduction does not play a role in the emergence of the dorsal fin primordium. Instead, the reduction occurs along with body growth after primordium formation, rather than through actively scrapping the non-fin forming region by inducing cell death. We also report that the emergence of specific mesenchymal cells and their proliferation promote dorsal fin primordium formation. Based on these results, we propose a revised hypothesis for median fin evolution in which the acquisition of de novo developmental mechanisms is a crucial evolutionary component of the discrete forms of median fins.

## Introduction

Fish, defined as vertebrates without tetrapods in this article, are characterized by unique appendages called fins in their morph^1^. Fish fins are classified into two groups: paired fins located on the ventral-lateral body trunk, and unpaired median fins situated on the body midline along the rostral-caudal axis^1,2^. The median fins, which allow fish to perform complex maneuvers in the water, show discrete forms (dorsal, anal, and caudal fins)^1–5^. These median appendages are of significant interest in evolutionary biology, in that they are considered to have evolved from a continuous midline fold-like structure called the median fin fold (MFF) in basal chordates (i.e., chordates other than the crown vertebrates in this article)^6–8^. In their development, fish median fins that are discrete forms in adulthood are thought to be derived from a continuous fold-like structure in the embryonic or larval stage, called the larval median fin fold (LMFF)^3,6,9–13^. It has been known that the evolutionary process of median fins may closely resemble the developmental process of median fins for a long time.

**Figure 1:**
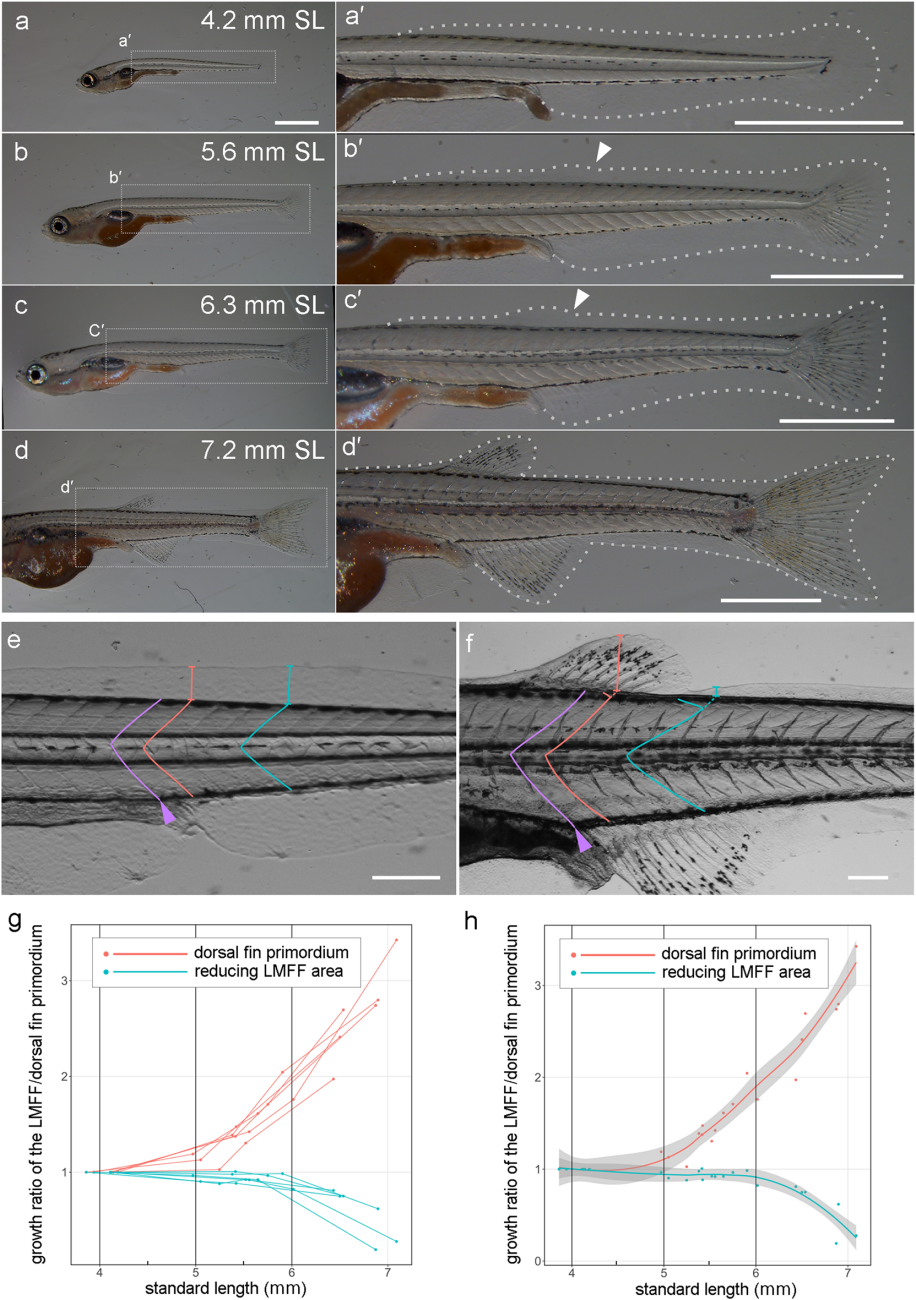
Morphological observation of dorsal fin development and LMFF reduction. (**a**-**d'**) Gross anatomy of median fin development at 4.2 mm (**a**,**a’**), 5.6 mm (**b**,**b’**), 6. 3 mm (**c**,**c’**), and 7.2 mm (**d**,**d’**). The right panels (**a’**,**b’**,**c’**,**d’**) are magnified views of the dashed rectangles in the left panes (**a**,**b**,**c**,**d**), respectively. White dashed lines in (**a’**,**b’**,**c’**,**d’**) indicate outlines of the LMFF. White arrowheads in (**b’**,**c’**) indicate protrusion sites of the LMFF. (**e**,**f**) Landmark and positions used for measuring the height of the LMFF. To examine the height of the LMFF/dorsal fin primordium at the same position during ontogeny, we used the somite boundary, which is located at the gut tube bending point (purple arrowhead) as a landmark (the first boundary: purple line). Then, we measured two somite boundaries: the next somite boundary from the first boundary (red line) for the future dorsal fin position and the fifth somite boundary (blue lines) for the fin-disappearing positions, respectively. (**g**,**h**) Transition of growth ratio of the LMFF/dorsal fin primordium. Each line in (**g**) indicates temporal transition of the same individual. (**h**) Local polynomial regression fit of (**g**). The 95% confidence intervals are indicated as grey areas in (**h**). Scale bars in (**a**) and those in (**a’**,**b’**,**c’**,**d’**,**e**,**f**) indicate 1 mm and 200 μm, respectively.

For the evolutionary and developmental process of the median fins, an influential hypothesis, the MFF hypothesis, has been proposed^1,2,6,8,12–16^. The MFF hypothesis assumes that the median fins evolved by reducing some positions in the MFF and retaining other parts of MFF^6,8,12–16^. In this widely accepted hypothesis, the process of fin fold reduction is considered to actively lead to the discontinuous individual median fins in fish. For example, in some fish species, tissue reduction has been reported in the inter-fin areas of the LMFF during development (Fig. 1a–d)^3,6,11,13,15,17–19^, and the median fin structures are raised at the remaining areas of the LMFF.

The median fins in teleosts are composed of the proximal cartilaginous skeleton (pterygiophores) and the distal dermal skeleton (fin rays)^17,20–22^. As described above, these median fins develop through dynamic morphological changes in the LMFF during their ontogeny^3,9–11,15,17–19^, but their developmental processes are still poorly understood at the cellular and molecular levels. For example, previous studies have proposed that the reduction of the LMFF occurs by apoptotic cell death^15,23^, and cell death at the LMFF has been reported in some studies^23–25^. However, cell death at the LMFF has been examined only during the embryonic stage when the median fin primordia have not yet appeared. In addition, migration of fin mesenchymal cells from the somite-derivatives into the LMFF occurs on two occasions; once during the early embryonic stage and again during the larval stage^26,27^. The mesenchymal cells that give rise to the median fin skeleton have been associated with the latter migration at around two weeks post-fertilization^27^. Since developmental mechanisms other than reduction may also contribute to median fin formation during post-embryonic development, the MFF hypothesis should be further examined and revisited from a developmental biological viewpoint.

In this study, we examined the transition from the LMFF into the dorsal fin in zebrafish at the levels of cellular and molecular developmental biology. We describe median fin morphogenesis in post-embryonic zebrafish larvae, and we also detected cell death and observed epithelial cell mass behavior in the reducing LMFF areas. We also describe mesenchymal cell behavior, including the distribution, differentiation, and proliferation of these cells, and assess the role of Fibroblast Growth Factor (FGF) signaling in dorsal fin primordia. Based on our results, we propose a revised hypothesis for median fin evolution from both developmental and phylogenetic perspectives.

## Results

### The timing of dorsal fin development differs from LMFF reduction

We first examined the initiation process of dorsal fin formation in the LMFF (Fig. 1). In 4.2 mm standard length (SL) zebrafish, the continuous LMFF appears to have no protrusions or outgrowths, implying that there is no fin primordial structure at the dorsal, caudal, and ventral midline (Fig. 1a,a’). When the larvae reach 5.6 mm SL, a small outgrowth was observed in the dorsal area of the LMFF (white arrowhead in Fig. 1b’). In 6.3 mm SL zebrafish larvae, the outgrowth continues to expand distally and along the rostral-caudal axis (white arrowhead in Fig. 1c’). By 7.2 mm SL, the dorsal fin primordium with fin rays is visible as described previously^18^. In 7.2 mm SL zebrafish larvae, the height of the LMFF appears reduced from the anterior side both before and behind the dorsal fin; the dorsal and caudal fins separated to form independent structures (Fig. 1d,d’). Taken together, we considered that the dorsal fin outgrowth at 5.6 mm SL may be the initial dorsal fin primordium.

To examine the detailed process of dorsal fin formation and LMFF reduction, we quantitatively analyzed the height of the LMFF at five day intervals during the transition process from 5 days post-fertilization (dpf) to 20 dpf (n = 6, Fig. 1e–h). We measured the height of the LMFF at the future dorsal fin-appearing position (red lines in Fig. 1e,f) and at the LMFF-disappearing position behind the dorsal fin (blue lines in Fig. 1e,f). The height of the LMFF at the dorsal fin position increased moderately as a protrusion between 4.5 and 5.0 mm SL and then started to protrude rapidly at around 5.0 mm SL (red dots and lines in Figs. 1g,h, S1a,b). The rapid increase continued until 7.0 mm SL. In contrast, the height of the LMFF at the presumptive disappearing position behind the dorsal fin was almost constant between 4.0 and 5.5 mm SL (blue dots and lines in Figs. 1g,h, S1a,b). Then, the height of that region started decreasing at around 5.5 mm SL before decreasing rapidly until 7.0 mm SL.

These findings suggest that the LMFF protrusion and outgrowth at the future site of the dorsal fin precedes the reduction of the LMFF. In other words, dorsal fin emergence may not be attributed to the process of LMFF reduction, but rather, to LMFF protrusion.

### Signals for apoptotic cell death were not detected during reduction of the LMFF

**Figure 2:**
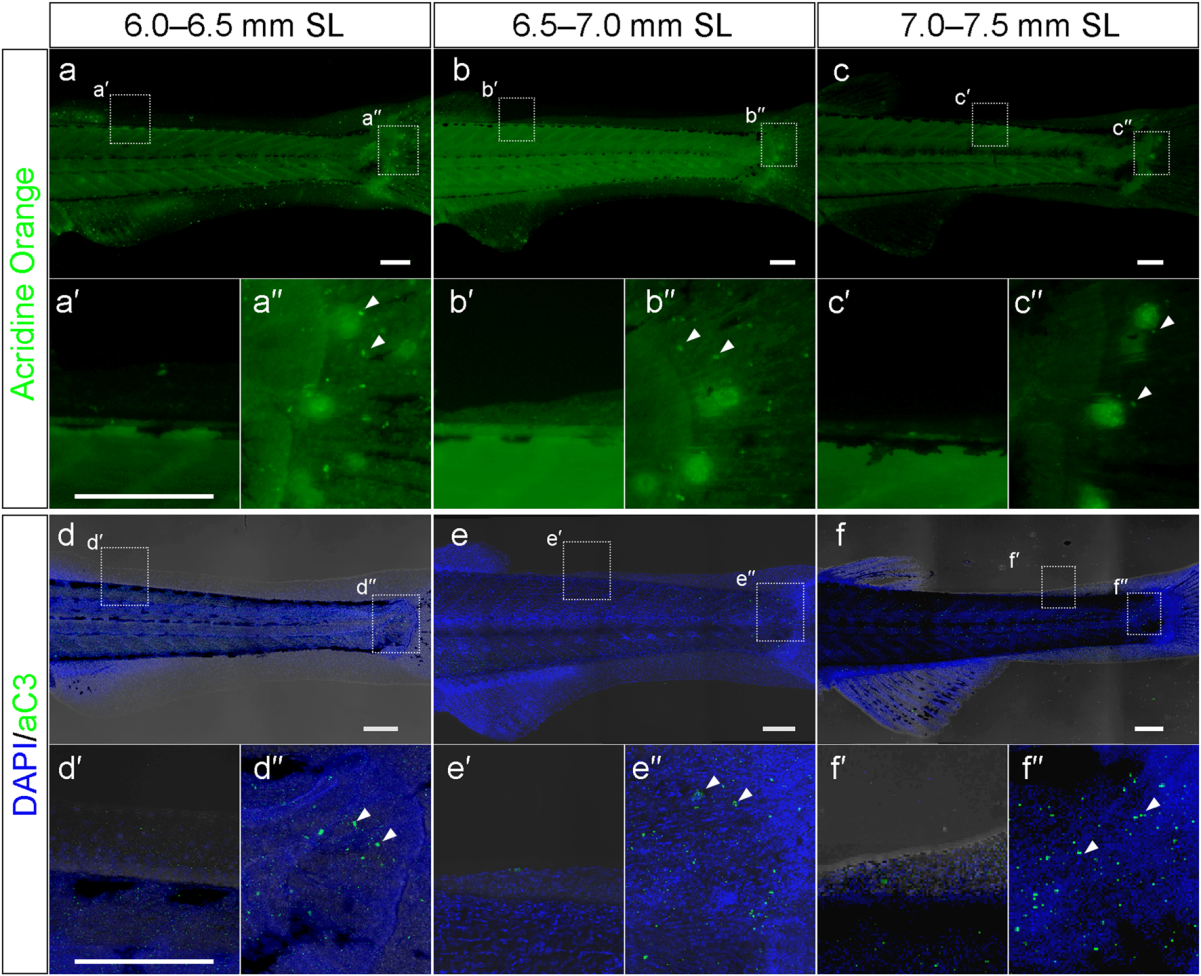
Apoptotic cell death in the reducing LMFF area. (**a**–**c’’**) Acridine orange staining in the reducing LMFF area (**a’**,**b’**,**c’**) and proximal part of the developing caudal fin region (**a”**,**b”**,**c”**) at 6.0–6.5 mm SL (**a**–**a”**), 6.5–7.0 mm SL (**b**–**b”**), and 7.0–7.5 mm SL (**c**–**c”**). The lower panels (**a’**,**a”**,**b’**,**b”**,**c’**,**c”**) are magnified views of the dashed rectangles in the upper panels (**a**,**b**,**c**), respectively. (**d**–**f”**) Expression pattern of active caspase 3 in the reducing LMFF area (**d’**,**e’**,**f’**) and proximal part of the developing caudal fin region (**d”**,**e”**,**f”**) area at 6.0–6.5 mm SL (**d**–**d”**), 6.5–7.0 mm SL (**e**–**e”**), and 7.0–7.5 mm SL (**f**–**f”**). The lower panels (**d’**,**d”**,**e’**,**e”**,**f’**,**f”**) are magnified views of the dashed rectangles in the upper panels (**d**,**e**,**f**), respectively. Arrowheads in (**a”**,**b”**,**c”**,**d”**,**e”**,**f”**) indicate examples of apoptotic cell death signals. Scale bars indicate 200 μm.

The reduction of the LMFF, which is a central component of the MFF hypothesis^6,8,12–16^, is considered to be associated with apoptotic cell death^15,23^. Since dorsal fin appearance precedes LMFF reduction, this raises the question of whether cell death in the LMFF is associated with dorsal fin formation. We therefore investigated cell death in associated with the LMFF reduction. First, we performed acridine orange staining, which is used to identify cell death in living specimens^28,29^, at the LMFF-reducing stage (6.0–6.5 mm SL, n = 6; 6.5–7.0 mm SL, n = 6; 7.0–7.5 mm SL, n = 5) (Fig. 2a–c”). We found no obvious signal for cell death in the reducing LMFF area in any samples (Fig. 2a,a’,b,b’,c,c’). However, some signals were detected in different areas, such as at the base of the caudal fin, indicating that the experimental procedure used for staining was fine (Fig. 2a’’, b’’, c’’). We further performed whole-mount immunohistochemistry with an anti-active caspase antibody at the LMFF-reducing stage (6.0–6.5 mm SL, n = 6; 6.5–7.0 mm SL, n = 5; 7.0–7.5 mm SL, n = 5) (Fig. 2d–f”)^30^. We detected some positive cells in a mesenchymal population at the base of the caudal fin (Figs. 2d’’,e’’,f’’, S2a,a’) and regenerating fin rays in the amputated caudal fin (Fig. S2b,b’, agreeing with previous report by Simões et al.^31^), indicating that the immunohistochemistry detected dying cells correctly. We observed very few positive signals in the reducing LMFF area (Fig. 2d’,e’,f’) in all samples, including specimens processed using another immunohistochemistry protocol that employed heat to activate the antigen (Fig. S2c,d,e).

These findings indicate that very little cell death occurs in the reducing LMFF area. In conclusion, cell death is not considered to play a major role in LMFF reduction and median fin segregation.

### Cell morphology and migration associated with LMFF reduction

**Figure 3:**
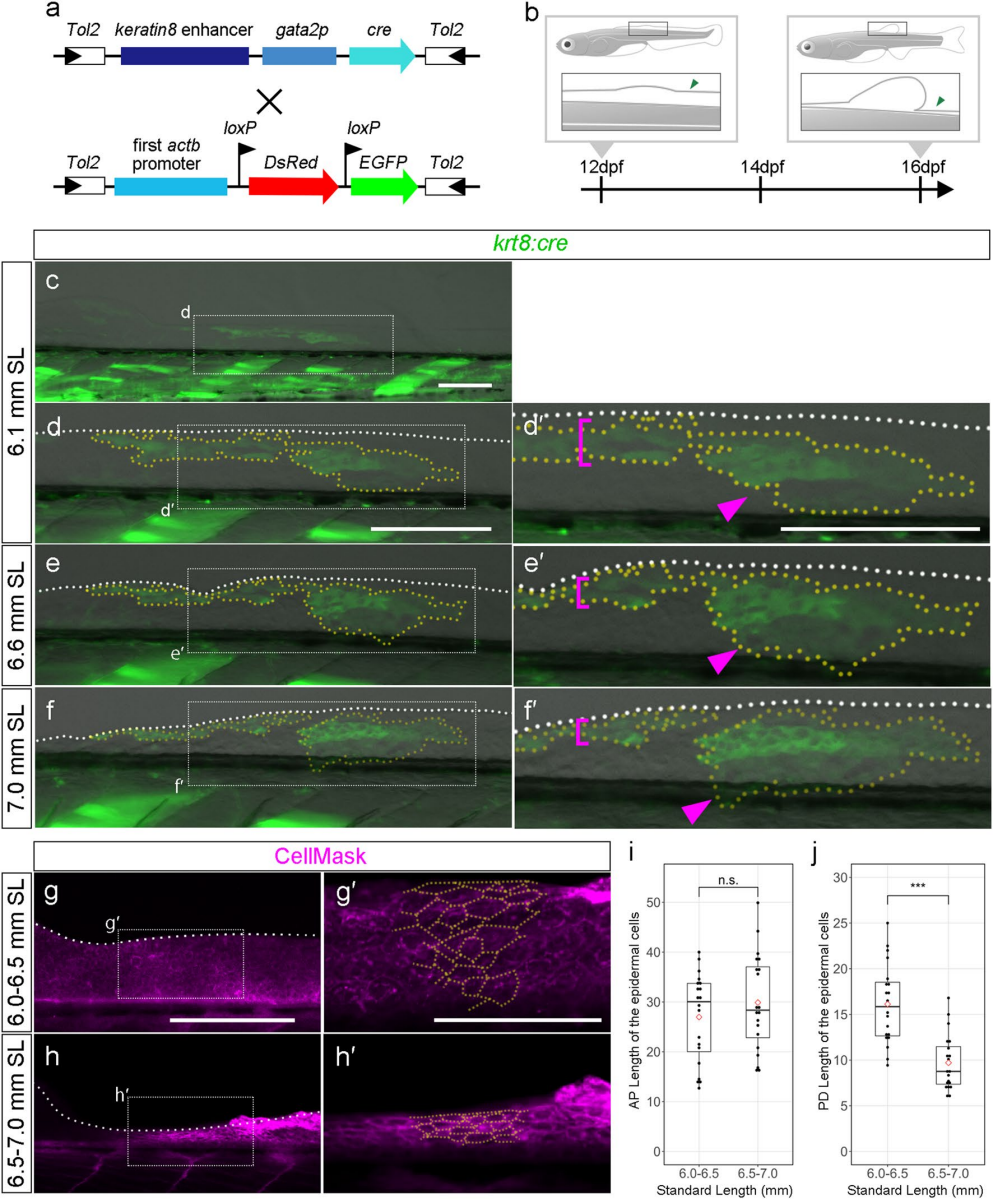
Cell-tracking analysis of the epithelial cells in the reducing LMFF area. (**a**) Schematic of the plasmid DNA construct used to generate the Tg. (**b**) Scheme of the Tg observation. (**c**–**f’**) GFP-positive labelled cells in the reducing LMFF area at 6.1 mm SL (**c**–**d’**), 6.6 mm SL (**e**–**e’**), and 7.0 mm SL (**f**–**f’**). The right panels (**d’**,**e’**,**f’**) are magnified views of the dashed rectangles in the left panes (**d**,**e**,**f**), respectively. White dashed lines in (**d-f’**) indicate outlines of the LMFFs. Yellow dashed lines indicate outlines of the EGFP-positive populations of epidermal cells. Magenta brackets in (**d’**,**e’**,**f’**) indicate EGFP-positive populations of epidermal cells experiencing proximo-distal shrinking. Magenta arrowheads in (**d’**,**e’**,**f’**) indicate EGFP-positive populations of epidermal cells migrating down to the trunk. (**g**–**h’**) Cell morphology and distribution in the reducing LMFF area at 6.0–6.5 mm SL (**g**–**g’**) and 6.5–7.0 mm SL (**h**–**h’**). Cell membrane visualized by CellMask. The right panels (**g’**,**h’**) are magnified views of the dashed rectangles in the left panels (**g,h**), respectively. Yellow dashed lines indicate outlines of the epidermal cells. (**i,j**) Boxplots of cell length along the AP and PD axis in the reducing LMFF area. Whiskers in (**i**) and (**j**) show maximum and minimum values within 1.5 times the interquartile range. Boxes show the median and 25th and 75th percentiles. The *P* value in (**i**) and (**j**) is the result of Brunner-Munzel test (*P* = 0.4407 and *P* = 8.34e-10). Scale bars in (**c,d,d’,g**) and that in (**g’**) indicate 200 μm and 100 μm, respectively.

The results above suggest that cellular behaviors other than cell death may play a role in LMFF reduction. We therefore examined epithelial cell migration and changes in cell morphology in LMFF reduction and performed in vivo cell-tracking analysis with an epidermal cell-specific *cre*-expressing vector (*krt8-p:cre*)^32^. We injected the *krt8-p:cre* vector into *Tg(actbp-loxP-DsRed-loxP-EGFP)*^33^ embryos (Fig. 3a) and observed EGFP-positive epidermal cells, which were distributed as mosaic patches in the LMFF of the injected specimens (Fig. 3c–f’). We traced the epidermal cell behavior from the stages when LMFF reduction started (12 dpf, 5.8–6.1 mm SL, n = 3), and specimens were observed every two days until 16 dpf, when larvae measured approximately 7.0 mm SL (Fig. 3b). Figure 3 shows a specimen in which two GFP-positive populations of epidermal cells can be observed in the reducing LMFF (yellow dotted area in Fig. 3d). During the observation period, GFP-positive populations narrowed along the proximal–distal axis by changing their morphology (magenta bracket in Fig. 3d’,e’,f’) and migrated down the trunk (magenta arrowheads in Fig. 3d’,e’,f’).

Furthermore, we used CellMask staining of the cell membrane to examine cell morphology and distributions more precisely in the reducing LMFF at the LMFF-reducing stage (6.0–6.5 mm SL, n = 4; 6.5–7.0 mm SL, n = 4) (Fig. 3g–h’). In the early stage of LMFF reduction (6.0–6.5 mm SL), the epidermal cells in the reducing LMFF were round. However, at the latter phase of the LMFF reduction (6.5–7.0 mm SL), the epidermal cells in the reducing LMFF were more narrowed along the proximal–distal axis (yellow dotted line in Fig. 3h,h’). Quantitative analysis showed that the epidermal cells in the reducing LMFF area remained their AP lengths and became shrunk along the PD axis (Fig. 3i,j). Focusing on the cell rows crossing the proximal–distal axis in the reducing LMFF, the thickness of the rows of cells were reduced as LMFF reduction progressed.

These results indicate that epidermal cells change shape and shrink when they migrate from the LMFF to the body trunk, suggesting that changes in cell morphology and migration of LMFF epidermal cells contribute to the process of LMFF reduction.

### Mesenchymal cell growth in the dorsal fin primordium

Our morphological observations (Fig. 1) and those of previous report^18^ also suggest that, rather than LMFF reduction, the protrusion and outgrowth of the LMFF is a key process in dorsal fin formation. Previous studies have shown that the mesenchymal cells are condensed at the future site of the dorsal fin^18^, and that somite-derived mesenchymal cells develop into dorsal fin skeletal elements^27^. These studies, however, did not show when mesenchymal cells emerge or the differentiation process of the mesenchyme. Thus, we investigated mesenchymal cell behavior during the formation of the dorsal fin primordium by using a reporter transgenic fish line.

**Figure 4:**
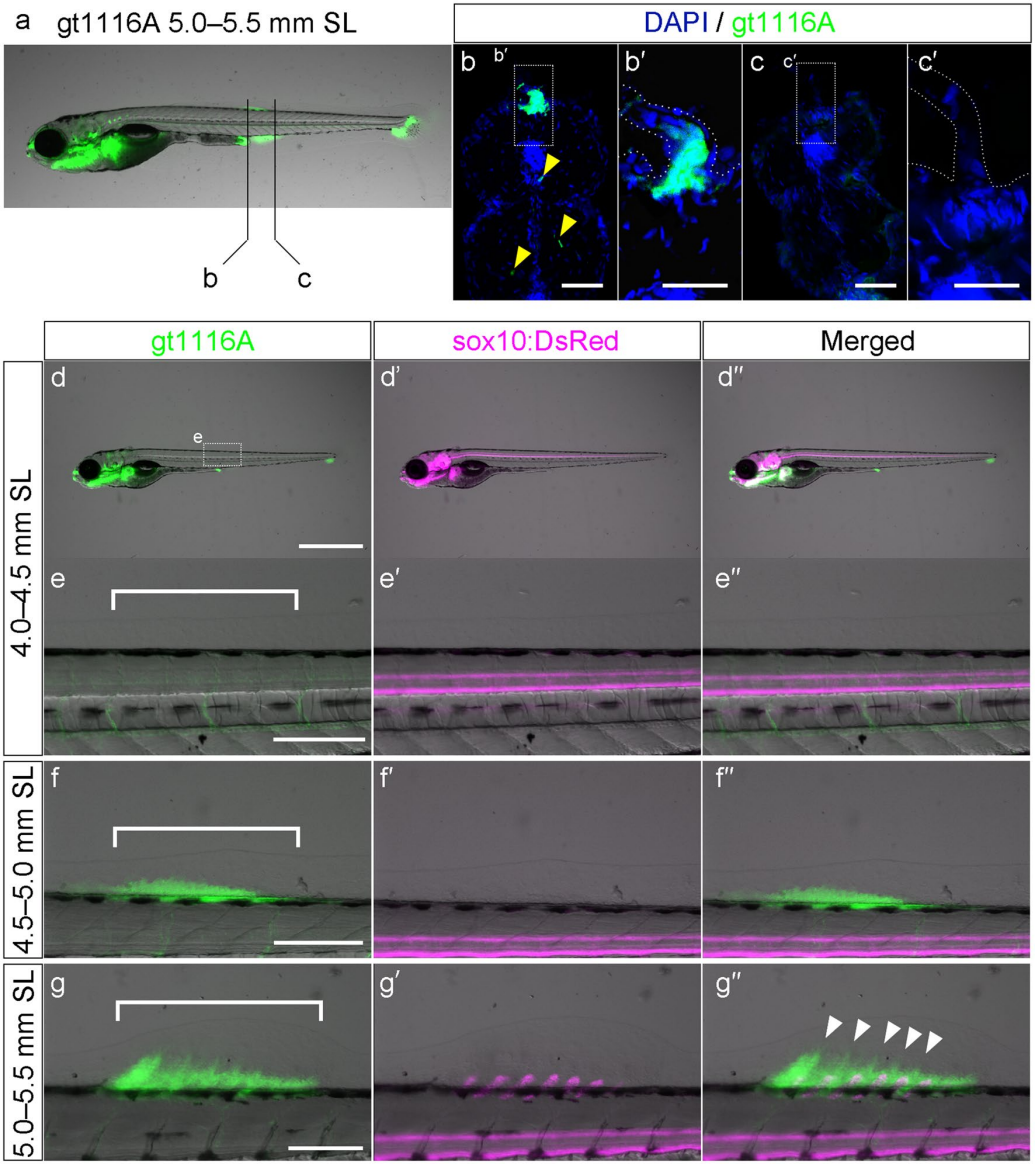
Expression pattern of UAS:EGFP in the *gt1116A* line and *sox10:DsRed* in the LMFF of double-transgenic zebrafish. (**a**–**c’**) Expression pattern of UAS:EGFP of the *gt1116A* line in the LMFF at 5.0–5.5 mm SL. Black lines in (**a**) indicate levels of the section shown in (**b,c**). Yellow arrowheads in (**b**) indicate blood vessels. Expression pattern of UAS:EGFP of the *gt1116A* line and *sox10:DsRed* in the LMFF of double-transgenic fish at 4.0–4.5 mm SL (**d**–**e”**), at 4.5–5.0 mm SL (**f**–**f”**), and 5.0–5.5 mm SL (**g**–**g”**). White brackets in (**e,f,g**) indicate future sites of the dorsal fins. White arrowheads in (**g”**) indicate distal expansion of the EGFP-positive mesenchymal cell population. Scale bars in (**b**–**c’**) and those in (**d,e,f,g**) indicate 50 μm and 200 μm, respectively.

We observed reporter expression in the *gt1116A* transgenic line (Fig. 4). In *gt1116A*, the gene-trapping *gal4* construct was integrated within the *prdm16* gene, and the UAS:EGFP reporter was found to be expressed in mesenchymal cell populations of the early pectoral fin bud^34,35^. In addition, UAS:EGFP in *gt1116A* has been reported to be expressed in other developing median fins, including the dorsal fin^35^. We first examined whether EGFP expression patterns of *gt1116A* are valid as a reporter of the fin mesenchyme in the developing dorsal fin primordium. The reporter EGFP was detected in median fin formation at the stage when the LMFF of the future dorsal fin site starts protruding (Fig. 4a; SL = 5.0–5.5 mm). Transverse sections showed UAS:EGFP expression distributed in cells of the middle at the LMFF protrusion, sandwiched by two outer layers of cells (presumably epidermal layers) where no signal was detected (Fig. 4b,b’). Mesenchymal cells in the LMFF of the inter-fin area between the dorsal fin and caudal fin showed no EGFP expression (Fig. 4c,c’). Several blood vessels in the trunk region showed EGFP expression, as reported in the orthologous gene in the mouse (yellow arrow heads in Fig. 4b)^36^, but vessel expression far from the fin primordium appears not to be related to the fin mesenchymal cell population. These findings confirmed that UAS:EGFP in the *gt1116A* line is expressed in the mesenchymal cells of the dorsal fin primordium.

We next assessed the expression pattern of the UAS:EGFP reporter in the *gt1116A* line in the transition from LMFF to dorsal fin primordium while observing chondrocytes by *sox10:DsRed* (4.0–4.5 mm SL, n = 6; 6.5–7.0 mm SL, n = 5; 7.0–7.5 mm SL, n = 5) (Fig. 4d–g’’). In 4.0–4.5 mm SL zebrafish, which are before the protrusion of the LMFF (Fig. 1h), no EGFP-positive cells were observed at the future site of the dorsal fin (white bracket in Fig. 4e). In 4.5–5.0 mm SL zebrafish, when the LMFF begins protruding (Fig. 1h), a mass of EGFP-positive cells was observed in the future site of the dorsal fin (white bracket in Fig. 4f). In 5.0–5.5 mm SL zebrafish with the LMFF outgrowth, the EGFP-positive mesenchymal cell population expanded distally (white bracket in Fig. 4g). The DsRed-positive cartilaginous elements, which become pterygiophores (basal elements of the dorsal fin skeleton), emerged at the lower part of the mesenchymal cell population (Fig. 4g’). In addition, because the site of the cartilage formation matched the distal expansion of the EGFP-positive mesenchymal cell population, these cells may give rise to the fin rays (white arrowheads in Fig. 4g’’). These findings suggest that EGFP-positive cells of the *gt1116A* line develop into dorsal fin skeletal elements, such as pterygiophores and fin rays. Thus, the dorsal fin-specific developmental mechanisms with mesenchymal cells, at least as defined by expression of *prdm16*, appeared simultaneously with the protrusion of the LMFF at 4.5–5.0 mm SL, and we define the protrusion with *prdm16*-positive mesenchyme as the initial dorsal fin primordium.

### Cell proliferation in dorsal fin mesenchyme

**Figure 5:**
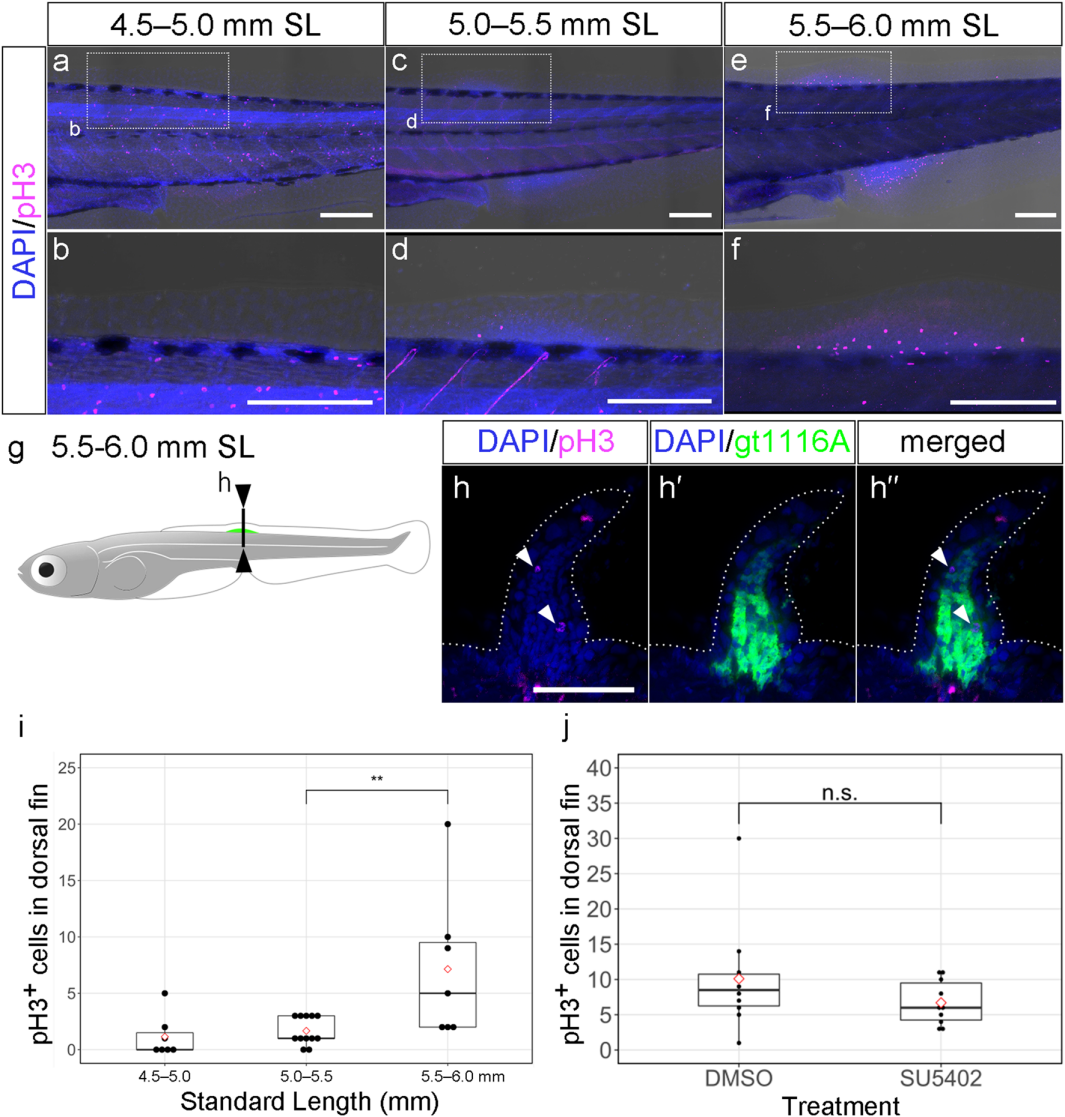
The expression pattern of phospho-histone-H3 in dorsal fin primordium. (**a**–**f**) Expression pattern of phospho-histone-H3 in the reducing LMFF area at 4.5–5.0 mm SL (**a**,**b**), 5.0–5.5 mm SL (**c**,**d**), and 5.5–6.0 mm SL (**e**–**f**). The lower panels (**b**,**d**,**f**) are magnified views of the dashed rectangles in the upper panels (**a**,**c**,**e**), respectively. (**g**,**h’’**) The expression pattern of UAS:EGFP of the *gt1116A* line and phospho-histone-H3 in dorsal fin primordium at 5.5–6.0 mm SL. Black lines in (**g**) indicate levels of the section shown in (**h**–**h’’**). Arrowheads in (**h**,**h”**) indicate phospho-histon-H3 signals. (**i**) Boxplots of phospho-histone-H3-positive cells in dorsal fin development along with the growth of the body. In (**i**), more than half of the 4.5–5.0 mm SL zebrafish samples, phospho-histone-H3-positive cells were not detect. Thus, we conducted statistical analysis only between 5.0–5.5 mm SL and 5.5–6.0 mm SL. (**j**) Boxplots of phospho-histone-H3-positive cells in dorsal fin development under SU5402 treatment. The proportions in (**i**) and (**j**) were calculated from the number of pH3-positive cells in dorsal fin primordium. Whiskers in (**i**) and (**j**) show maximum and minimum values within 1.5 times the interquartile range. Boxes show the median and 25th and 75th percentiles. The *P* value in (**i**) and (**j**) is the result of Brunner-Munzel test (*P* = 0.009701) and the result of Welch's *t* test (*P* = 0.2272), respectively. Scale bars in (**a**–**f**) and those in (**h**) indicate 200 μm and 50 μm, respectively.

An analysis of the *gt1116A* line suggested that the development of the dorsal fin primordial mesenchyme is associated with the protrusion of the LMFF, which raises the question of whether cell proliferation in the fin mesenchyme contributes to dorsal fin primordium development. To identify the distribution of proliferating cells in the dorsal fin primordium, we performed immunohistochemistry with an anti-phospho histone H3 (pH3) antibody^30,35^. In 4.5–5.0 mm SL zebrafish (n = 7) (Fig. 5a,b), a few pH3-positive cells were detected within the dorsal fin primordium in some samples (n = 3/7). In many samples (n = 10/12) of 5.0–5.5 mm SL zebrafish, pH3-positive cells were detected, though the number of positive cells was still few (Fig. 5c,d). In 5.5–6.0 mm SL zebrafish, all samples (n = 7/7) showed many pH3-positive cells in the dorsal fin primordium (Fig. 5e,f). Figure 5g–h’’ shows that pH3-positive cells (White arrowheads in Fig. 5h,h’’) were located in the EGFP-positive cells of the *gt1116A*, indicating that these are mesenchymal cells in the dorsal fin primordium (Fig. 5g–h’’). Quantitative analysis confirmed that the number of pH3-positive cells in the dorsal fin primordium increases along with body growth from 4.5 to 6.0 mm SL (Fig. 5i). Interestingly, the stage when the proliferation of the mesenchymal cells starts increasing corresponds to the stage when the height of the dorsal fin primordium starts increasing rapidly during the process of outgrowth (5.0 mm SL, Fig. 1h). This suggests that mesenchymal cell proliferation plays a role in the outgrowth of the dorsal fin primordium.

In some fish, such as sharks and cichlids, it has been shown that FGF signaling is involved in dorsal fin development^19,37^. In larval zebrafish, it has also been shown that FGF signaling plays a role in the early stages of LMFF development^10^. Therefore, we sought to investigate the role of FGF signaling in the cell proliferation of the dorsal fin primordium. SU5402 is a chemical inhibitor of Fgfr that has been reported to specifically inhibit the kinase activity of nearly all types of Fgfr^10,38,39^. Treatment for three days with SU5402 at 5.0–5.5 mm SL resulted in no significant effect on cell proliferation in the early dorsal fin primordium (Figs. 5j, S3a). These findings suggest that FGF signaling may not play a role in the early proliferation of dorsal fin mesenchymal cells (5.0–6.0 mm SL). In anal fins, on the other hand, SU5402 treatment at 5.0–5.5 mm SL inhibited cell proliferation (Fig. S3b). It is possible that FGF signaling may play a role in the latter stages of median fin development, since anal fin development precedes dorsal fin development. Furthermore, SU5402 treatment also did not affect the height of the dorsal fin primordium when zebrafish were between 5.0 and 6.0 mm SL (Fig. S3c, d). Taken together, these findings suggest that FGF signaling does not play a role in at least the initial protrusion process of dorsal fin primordium.

## Discussion

### Developmental processes critical to median fin formation

The discrete median fins in modern fish evolved from the continuous MFF in the basal chordate, resembling the developmental process of the median fins derived from the LMFF^6,8,12,13,15^. Based on this resemblance, the MFF hypothesis assumes that reduction of the fin fold is a key evolutionary process in the segregation of independent median fins in vertebrate phylogeny from the view of recapitulation theory^6,8,14–16^. This is because of the classical assumption that fish median fin development is caused by the simple reduction of the LMFF. However, our present findings revealed that the reduction of the LMFF occurred after the LMFF protrusion at the future site of dorsal fin formation (Fig. 1). Furthermore, although previous studies supposed that LMFF reduction is caused by apoptosis along the inter-fin areas of the LMFF^15,23,25^, our findings suggest that cell death did not play a role in LMFF reduction (Fig. 2). So, how does the reduction of the LMFF occur during zebrafish ontogeny? Our cell-tracking analyses revealed that cell behaviors, such as narrowing proximo-distally and migrating to the body trunk region (Fig. 3), are responsible. During the period when LMFF reduction occurs, zebrafish larvae increase vigorously in body size (both length and width). It is therefore likely that LMFF epithelial tissue may be involved in this expansion of the body surface, shrinking along the proximal–distal axis and moving to the trunk region. It is noteworthy that this collective migration of epidermal cells is seen at a relatively late stage, i.e., when the dorsal fin primordium has already protruded and the LMFF has reduced. This suggests that LMFF reductions do not drive the emergence of median fin primordia. We postulate that regression or degradation of the supporting material in the LMFF structure, such as actinotrichia or extracellular matrix complex with laminin^40–43^, reoriented epithelial migration into the trunk at the inter-fin area. Further molecular developmental biological studies are required to reveal how this collective epithelial cell movement occurs. Together with our cell behavioral analyses, we propose that the reduction of the LMFF is not associated with dorsal fin formation. Instead, the LMFF reduction appeared to occur together with an increase in body size. In other words, other cellular and developmental processes could be responsible for dorsal fin formation during ontogeny.

What kinds of cellular developmental mechanisms play a role in dorsal fin formation? From examining the appearance of the dorsal fin primordium, our findings show that the dorsal fin primordial mesenchyme, which expresses UAS:EGFP in the *gt1116A* line and develops into the adult fin skeleton (Fig. 4), emerges simultaneously with the LMFF protrusion (Fig. 1). Due to *gt1116A* trapping *prdm16*, we suggest that developmental mechanisms that specify the mesenchymal cell population expressing developmental genes, such as *prdm16*, play a role in dorsal fin primordium formation. The rapid increase in the LMFF height in the dorsal fin primordium at around 5.0 mm SL is correlated with the appearance of proliferation of mesenchymal cells (Fig. 5c–h), suggesting that this cell proliferation promotes the outgrowth of the dorsal fin primordium. In addition, cell migration may also contribute to dorsal fin development along with cell proliferation. In 4.5–5.0 mm SL zebrafish, most samples did not have cell proliferation signals as detected by anti-pH3 antibody in the dorsal fin primordium. In 5.0–5.5 mm SL zebrafish, the number of pH3-positive cells was relatively small compared to the total number of dorsal fin primordial mesenchymal cells (Fig. 5i). Previous studies have shown that fin osteoblasts are derived from a secondary source of somite-derived cells, and not from cells present in the LMFF before hatching^26,27^. We suggest that migration of the somite-derivative cells into the fin primordium at the initiation of the protrusion stage in particular, as well as during the latter outgrowth stage, contributes to the increase of mesenchymal cell mass in the dorsal fin primordium. In addition, although FGF signaling contributes to initial LMFF formation^10^, our pharmacological assays showed that this mechanism does not play a role in the early outgrowth phases of dorsal fin primordia (Figs. 5j and S3c,d). This discrepancy in molecular signaling mechanisms between early induction of the LMFF and later outgrowth at the dorsal fin primordium suggest that the developmental modules associated with the dorsal fin primordium are independent of those involved in LMFF formation. Previous studies have shown that some zebrafish mutants with malformed LMFFs, which are bubbly or in which the edge of the fin fold is collapsed, develop normal adult median fins^44^. This evidence supports our inference that the developmental module of the dorsal fins behaves independently from the LMFF developmental module.

### Revision of the evolutionary events essential for the acquisition of median fins

Our ontogenetical evidence does not support the MFF hypothesis. Thus, we would revise the phylogenetic assumptions concerning the critical developmental mechanisms that are responsible for evolving the discrete form of median fins.

The MFF hypothesis predicts that discrete median fins appeared by acquiring a mechanism to transition from the LMFF to median fins, based on the premise that the fish LMFF is homologous to the plesiomorphic MFF of basal chordates. The lower Cambrian basal chordates *Haikouichthys* and *Myllokunmingia* had numerous anatomical structures that inform the taxonomic affinity of stem vertebrates^45–47^. For example, they had an MFF, but previous studies lacked sufficient evidence to confirm the presence of endoskeletal elements^46–48^. Thus, it was naturally assumed that animals in the lineage from basal chordates to early vertebrates continuously possessed MFFs, and that fish LMFFs are homologous to these MFFs^15,48,49^. Addition to this premise, the MFF hypothesis presumes that median fin development occurred by a reduction of the inter-fin region based on morphological observations of ontogeny in living fish^2,6,8,12–14^. From this phylogenetic and ontogenetic perspective, the MFF hypothesis implies that the median fins evolved from ancestral MFFs by reduction of the LMFF^6,8,12–16^. However, our detailed morphological and molecular observations on the ontogenetic process from LMFFs to median fins suggest that median fin-associated developmental modules behave independently of the LMFF developmental module. We hypothesize that acquiring the developmental process of LMFF reduction is not the main contributor to the evolution of median appendages. Rather, acquiring de novo developmental mechanisms in which a mesenchymal cell mass invades into the LMFF and expresses a specific genetic program for developing adult median fins is the key evolutionary component of the discrete form of median fins. Some fishes in the basal lineage, such as chondrichthyans and cyclostomes, have median fins with specific skeletal elements which are at least partially homologous to those of teleost median fins^50,51^. Thus, the median fins in chondrichthyans and cyclostomes might therefore share the de novo developmental mechanisms with teleost median fins. This assumption reinforces our argument about the origin of median fin. However, how those median fins develop from LMFF is still unclear, and further investigations of the developmental processes in various fish, such as cyclostomes and chondrichthyans, will help to test our hypothesis of the phylogenetic process of acquiring median fins and will further illuminate the origin of fin skeletal components such as fin rays and pterygiophores.

### Differences between the development of median and paired fins

The evolutionary origin of paired fins is also considered to be related to median fin evolution. Paired fins in most fish exhibit the basic skeletal configuration seen in median fins, with a basal endoskeleton and associated fin rays^2,4^. One influential idea assumes that paired fins arose by co-opting of the genetic patterning modules established during median fin evolution^17,52,53^. Indeed, gene expression studies in paired and median fins have identified a similar pattern in the expression of developmental genes, such as the nested expression of Hox genes^19,37,52–54^. We found that *prdm16* is expressed in dorsal fin mesenchyme by observations of EGFP expressions in the *gt1116A* line, and this gene is also expressed in pectoral fin mesenchyme at the early embryonic stage^35,55,56^. Our and previous studies indicate that *prdm16*-positive mesenchymal cells differentiate into skeletal elements in both pectoral and dorsal fins^35^. This gene expression pattern suggests that median and paired fins share developmental mechanisms and supports the hypothesis that the developmental mechanism of mesenchymal cells was co-opted from median fins to paired fins. However, although FGF signaling plays an essential role in pectoral fin buds at the early embryonic stage^57,58^, our pharmacological assays with an FGF signaling inhibitor showed no apparent effect on the early development of dorsal fins (Figs. 5j, S3c,d). Based on these similarities and differences, our hypothesis of paired fin origins holds that paired fins arose by partial co-option of ancestral genetic modules that were first present in median fins.

## Methods

### Zebrafish strains

The following transgenic zebrafish lines were used in this study: *gt1116A* (*gSAIzGFFD1116A:Gal4FF;UAS:EGFP*, trapping the *prdm16* gene)^35^ and *sox10:DsRed*^59,60^. To generate *krt8-p:Cre* (*keratin8 enhancer:gata2 promoter:Cre*) transgenic fish, we injected the *krt8-p:Cre* plasmid with *Tol2 transposase* mRNA into *actb-p:loxp-RFP-loxp-GFP* eggs^33^. To prepare *krt8-p:Cre*, genomic DNA fragments were isolated by using PCR (Fwd: GAG TCG ACG CCT TTG AAA TGT AAA AGC TCA, Rev: ATC CTG CCT TGT GTG TTT TCT GTC TTG T)^32^ and integrated with a downstream *Cre* gene into the *tol2* plasmid^61^.

Zebrafish were housed at 28 °C under light for 14 h^62^, and the standard length (SL) of individuals was measured^18^. To indicate the body size of individuals, we used SL instead of the date of development, since zebrafish of the same age often have different body sizes^18^. All experimental animal care procedures were conducted in accordance with institutional and national guidelines and regulations, and were approved by the Tohoku University Animal Research Committee (Permit Number: 2019LsA-022). The study was carried out in compliance with the ARRIVE guidelines.

### Observation of zebrafish Tg

Zebrafish larvae less than 6.0 mm SL were anesthetized with 0.025% MS222/E3 and then embedded in 2% methylcellulose/E3 on a slide-glass, dropping 0.125% MS222/E3. Specimens more than 6.0 mm SL were anesthetized with 0.025% MS222/E3 and placed on a 1% agarose-gel/E3. These zebrafish were observed under a microscope (Leica M205 FA) and photographed (Leica DFC 360 FX). Images were obtained and analyzed with Leica LAS-AF, LAS-X, and Adobe Photoshop CS6 after observation, and the larvae were immediately transferred to a small case filled with system water and awakened with sprayed water.

### LMFF/dorsal fin primordium height measurements and growth ratio calculations

LMFF/dorsal fin primordia heights were measured as described below. LMFF/dorsal fin primordia were observed under a microscope (Leica M205 FA) and photographed (Leica DFC360 FX). To reduce variance in the measured length from the photographs, image capture and preparation were repeated three times. LAS AF Lite was used to measure the standard length, fin primordium, and LMFF from the photographs. The mean of three measurements was used in the analysis.

To examine the height of the LMFF/dorsal fin primordium at the same position during ontogeny, we used the somite boundary, which is located at the flexion of the gut tube (purple arrowhead in Fig. 1e,f) and was used as a landmark (the first boundary, purple line in Fig. 1e,f). Then, we measured two somite boundaries: the next somite boundary (red line in Fig. 1e,f) and the fifth somite boundary from the first boundary (blue lines in Fig. 1e,f). In larvae less than approximately 6.0 mm SL, which have “V”-shaped somites, we measured the height of the LMFF/dorsal fin primordium from the intersection of the somite boundary and the border of the trunk to the distal tip of the dorsal fin primordium/LMFF (red and blue lines in Fig. 1e). In larvae longer than about 6.0 mm SL, which have “W”-shaped somites, we measured the height of the LMFF/dorsal fin primordium from the intersection (Fig. 1f) of the extension line of the middle part of this somite boundary (dashed red and blue line in Fig. 1f) and the border of the trunk to the distal tip of the dorsal fin primordium/LMFF (red and blue lines in LMFF in Fig. 1f).

The growth ratio of the LMFF/dorsal fin primordium heights on N dpf (N = 5, 10, 15, 20) was calculated as (LMFF/dorsal fin primordium height on N dpf)/(LMFF/dorsal fin primordium height on 5 dpf).

### Immunohistochemistry

Whole mount and section immunohistochemistry were used to detect cell death (anti-Caspase-3, #ab13847, Abcam), mitosis (Mouse monoclonal anti-pH3, #9706, Cell Signaling Technology) and anti-green fluorescent protein (GFP, #A11120 and #A6455, Invitrogen).

Whole mount immunohistochemistry was performed as described previously ^30,34^ with minor modifications. Zebrafish larvae at a suitable stage were collected by observation under a microscope (Leica M165C), fixed with 4% PFA/PBS, dehydrated with methanol/PBT (0.1% Tween-20 in PBS) and stocked in absolute methanol at -20 °C. Samples were rehydrated with methanol/PBT, permeabilized with 0.5% TritonX-100, blocked with blocking buffer (2% BSA, 1% goat serum, and 1% DMSO in PBT) and stained with a 1:1000 dilution of anti-Caspase3 antibody or 1:500 dilution of anti-pH3 antibody in blocking buffer. Samples were then washed five times in PBT, blocked with blocking buffer and incubated with a 1:500 dilution of secondary antibody (anti-mouse Alexa Flour 488 goat anti-mouse IgG, #A11001, Invitrogen). After washing five times in PBT, samples were stained with a 1:250,000 dilution of DAPI(4’,6-Diamidino-2-phenylindole dihydrochloride,#049-18,801,Wako) for 30 min and were washed 3 times in PBT. The head and abdomen were removed from the samples to prepare them for observation. The samples were placed on a glass slide, covered with a coverslip, lightly pressed, and observed under a confocal microscope (Leica TCS SP5 II). Optionally, the heating method after rehydration was performed as described previously^63^.

Section immunohistochemistry staining was performed as described previously ^59^ with minor modifications. Frozen sections of fixed zebrafish were prepared with a cryostat (Leica CM3050 S). The sections were washed three times in TNT for 5 min. After blocking with 1.5% blocking reagent (#11,096,176,001, Roche) in TNT for 1 h, the sections were incubated overnight at 4 °C with a 1:200 dilution of anti-GFP antibody and anti-pH3 antibody in blocking regent. Samples were then washed three times in TNT and incubated with a 1:500 dilution of secondary antibody (anti-mouse Alexa Flour 488 goat anti-mouse IgG, #A11001, Invitrogen) and a 1:100,000 dilution of DAPI for 1 h. After washing four times in TNT, samples were sealed by VECTASHIELD (#H-1000, Vector Laboratories). The samples were then observed under a confocal microscope (Leica TCS SP5 II).

### Acridine orange staining

Acridine orange (AO; #A6014-10G, Sigma) was used to identify cell death including apoptosis^28,29^ following the method of Freitas et al.^27^. Embryos were incubated in the dark in 0.5 μg/ml AO in PBS at room temperature for 30 min and rinsed three times in fish water for 10 min. Samples were observed under a microscope (Leica M205 FA) and photographed (Leica DFC 360 FX) under UV fluorescence.

### Fin amputation

Fish were anesthetized with 0.025% MS222/E3. Caudal fins were amputated with a scalpel. The amputation site was parallel to the dorsal–ventral axis at the tip of a non-segmented fin ray connected to the uroneural.

### CellMask staining

CellMask (#C10046, Invitrogen) was used to stain cell membranes following the method of Jia et al.^65^. Living embryos were incubated in 5 μg/ml CellMask in E3 at room temperature for 3 h in the dark and rinsed three times in fish water for 10 min. Specimens were anesthetized with 0.025% MS222/E3 and placed on a low melting agarose gel (NuSieve™ GTG™ Agarose, #50,081, Lonza). These zebrafish were observed under a confocal microscope (Leica TCS SP5 II) and irradiated with a 633 nm laser.

### SU5402 treatment

Inhibition of signaling through FGF receptors was performed with the lipophilic reagent SU5402 (#572,630, CalBiochem)^39^. Embryos were incubated in the dark at 28.5 °C with 20 μM SU5402 in fish water, prepared from 5 mM SU5402 stock solution in DMSO^10,64^. Control embryos were incubated with the corresponding amount of DMSO. After treatment, some samples were fixed in 4% PFA in PBS for 16–24 h, and cell mitosis was detected by immunohistochemistry. In the other samples, the height of the fin primordium was examined under a microscope (Leica M205 FA) and photographed (Leica DFC 360 FX).

### Statistical analysis

Scatter plots, which show the transition of the height of the LMFF/dorsal fin primordium, and box plots, which the length of the epidermal cells in reducing LMFF area and show the number of proliferating cells in the median fin primordium, were generated with the R (https://www.r-project.org/) package ggplot2. The local polynomial regression fit is shown in Figs. 1h, S1b, which shows the transition of the height of the LMFF/dorsal fin primordium and was obtained using the loess method. The local polynomial regression lines and R^2^ values were computed in R (ggplot, method = lm). For quantitative analysis of the length of the epidermal cell layer in reducing LMFF area, we select 5 cells from each specimens at random and Brunner-Munzel test was performed in R using the brunner.munzel.test function. For quantitative analysis of the number of proliferating cells in the median fin primordium, Brunner-Munzel test and Welch’s *t*-test were performed in R using the brunner.munzel.test and the t.test function. For quantitative analysis of the pharmacological effects on the height of LMFF/dorsal fin primordium, analysis of covariance (ANCOVA) between groups was computed in R using the ANOVA function.

## Supplementary Information

Supplementary Information.

## Data Availability

The datasets used and/or analyzed in the present study are available from the corresponding author on reasonable request.

## References

[1] Larouche O, Zelditch ML, Cloutier R (2019). A critical appraisal of appendage disparity and homology in fishes. Fish Fish..

[2] Romer AS, Parsons TS (1986). The Vertebrate Body.

[3] Bemis WE, Grande L (1999). Development of the median fins of the North American paddlefish *(Polyodon spathula*), and a reevaluation of the lateral fin-fold hypothesis. Mesozoic Fishes.

[4] Kardong KV (2019). Vertebrates : Comparative Anatomy, Function, Evolution.

[5] Helfman GS, Collette BB, Facey DE, Bowen BW (2009). The Diversity of Fishes : Biology, Evolution, and Ecology.

[6] Goodrich E (1930). Studies on the Structure and Development of Vertebrates.

[7] Nishino A, Satoh N (2001). The simple tail of chordates: Phylogenetic significance of Appendicularians. Genesis.

[8] Thacher J (1877). Median and paired fins, a contribution to the history of vertebrate limbs. Trans. Connect. Acad..

[9] Dane PJ, Tucker JB (1985). Modulation of epidermal cell shaping and extracellular matrix during caudal fin morphogenesis in the zebra fish Brachydanio rerio. J. Embryol. Exp. Morphol..

[10] Abe G, Ide H, Tamura K (2007). Function of FGF signaling in the developmental process of the median fin fold in zebrafish. Dev. Biol..

[11] Suzuki T (2003). Differentiation of chondrocytes and scleroblasts during dorsal fin skeletogenesis in flounder larvae. Dev. Growth Differ..

[12] Balfour FM (1881). On the development of the skeleton of paired fishes of Elasmobranchii. Proc. Zool. Soc. London.

[13] Balfour FM (1878). A monograph on the development of elasmobranch fishes.

[14] Cole NJ, Currie PD (2007). Insights from sharks: Evolutionary and developmental models of fin development. Dev. Dyn..

[15] Stewart TA, Bonilla MM, Ho RK, Hale ME (2019). Adipose fin development and its relation to the evolutionary origins of median fins. Sci. Rep..

[16] Mivart, S. G. *Notes on the fins of Elasmobranchs, with considerations on the Nature snd Homologues of Vertebrate Limbs*. (Longmans, Green, Reader, and Dyer, 1879).

[17] Mabee PM, Crotwell PL, Bird NC, Burke AC (2002). Evolution of median fin modules in the axial skeleton of fishes. J. Exp. Zool..

[18] Parichy DM, Elizondo MR, Mills MG, Gordon TN, Engeszer RE (2009). Normal table of postembryonic zebrafish development : Staging by externally visible anatomy of the living fish. Dev. Dyn..

[19] Freitas R, Zhang GJ, Cohn MJ (2006). Evidence that mechanisms of fin development evolved in the midline of early vertebrates. Nature.

[20] Eaton TH (1945). Skeletal supports of the median fins of fishes. J. Morphol..

[21] Lindsey CC (1955). Evolution of meristic relations in the dorsal and anal fins of teleost fishes. Trans. Roy. Soc. Can. Sect..

[22] Bird NC, Mabee PM (2003). Developmental morphology of the axial skeleton of the zebrafish, *Danio rerio* (Ostariophysi: Cyprinidae). Dev. Dyn..

[23] Cole LK, Ross LS (2001). Apoptosis in the developing zebrafish embryo. Dev. Biol..

[24] Abraham, N. Role of Programmed Cell Death in Defining Zebrafish Development. Faculty Dissertations. 65. St. John’s University, New York, USA (2004).

[25] Zuzarte-Luís, V. and Hurlé, J. Apoptosis in Fin and Limb Development. in *Fins into Limbs: Evolution, Development, and Transformation.* 103–108. (University of Chicago Press, 2008). doi:10.7208/9780226313405-009.

[26] Lee RTH, Knapik EW, Thiery JP, Carney TJ (2013). An exclusively mesodermal origin of fin mesenchyme demonstrates that zebrafish trunk neural crest does not generate ectomesenchyme. Dev..

[27] Ho Lee RT, Thiery JP, Carney TJ (2013). Dermal fin rays and scales derive from mesoderm, not neural crest. Curr. Biol..

[28] Hammerschmidt M (1996). dino and mercedes, two genes regulating dorsal development in the zebrafish embryo. Development.

[29] Freitas R, Zhang GJ, Cohn MJ (2007). Biphasic Hoxd gene expression in shark paired fins reveals an ancient origin of the distal limb domain. PLoS One.

[30] Sousa S (2011). Differentiated skeletal cells contribute to blastema formation during zebrafish fin regeneration. Development.

[31] Simões MG (2014). Denervation impairs regeneration of amputated zebrafish fins. BMC Dev. Biol..

[32] Gong Z (2002). Green fluorescent protein expression in germ-line transmitted transgenic zebrafish under a stratified epithelial promoter fromKeratin8. Dev. Dyn..

[33] Yoshinari N, Ando K, Kudo A, Kinoshita M, Kawakami A (2012). Colored medaka and zebrafish: Transgenics with ubiquitous and strong transgene expression driven by the medaka β-actin promoter. Dev. Growth Differ..

[34] Asakawa K (2008). Genetic dissection of neural circuits by Tol2 transposon-mediated Gal4 gene and enhancer trapping in zebrafish. Proc. Natl. Acad. Sci. U.S.A.

[35] Yoshida K, Kawakami K, Abe G, Tamura K (2020). Zebrafish can regenerate endoskeleton in larval pectoral fin but the regenerative ability declines. Dev. Biol..

[36] Craps S (2021). Prdm16 supports arterial flow recovery by maintaining endothelial function. Circ. Res..

[37] Höch R, Schneider RF, Kickuth A, Meyer A, Woltering JM (2021). Spiny and soft-rayed fin domains in acanthomorph fish are established through a BMP-gremlin-shh signaling network. Proc. Natl. Acad. Sci. U.S.A.

[38] Fürthauer M, Reifers F, Brand M, Thisse B, Thisse C (2001). Sprouty4 acts in vivo as a feedback-induced antagonist of FGF signaling in zebrafish. Development.

[39] Mohammadi M (1997). Structures of the tyrosine kinase domain of fibroblast growth factor receptor in complex with inhibitors. Science.

[40] Carney TJ (2010). Genetic analysis of fin development in zebrafish identifies furin and Hemicentin1 as potential novel fraser syndrome disease genes. PLoS Genet..

[41] Kuroda J, Itabashi T, Iwane AH, Aramaki T, Kondo S (2020). The physical role of mesenchymal cells driven by the actin cytoskeleton is essential for the orientation of collagen fibrils in zebrafish fins. Front. Cell Dev. Biol..

[42] Kuroda J, Iwane AH, Kondo S (2018). Roles of basal keratinocytes in actinotrichia formation. Mech. Dev..

[43] Webb AE (2007). Laminin α5 is essential for the formation of the zebrafish fins. Dev. Biol..

[44] Van Eeden FJM (1996). Genetic analysis of fin formation in the zebrafish, Danio rerio. Development.

[45] Miyashita T (2019). Hagfish from the Cretaceous Tethys Sea and a reconciliation of the morphological-molecular conflict in early vertebrate phylogeny. Proc. Natl. Acad. Sci. U.S.A.

[46] Shu DG (2003). Head and backbone of the Early Cambrian vertebrate Haikouichthys. Nature.

[47] Shu DG (1999). Lower Cambrian vertebrates from south China. Nature.

[48] Zhang XG, Hou XG (2004). Evidence for a single median fin-fold and tail in the Lower Cambrian vertebrate, Haikouichthys ercaicunensis. J. Evol. Biol..

[49] Schaeffer B (1987). Deuterostome monophyly and phylogeny. Evol. Biol..

[50] Ota KG, Fujimoto S, Oisi Y, Kuratani S (2013). Late development of hagfish vertebral elements. J. Exp. Zool. Part B Mol. Dev. Evol..

[51] Janvier, P. Homologies and evolutionary transitions in early vertebrate history. In *Major Transitions in Vertebrate Evolution* 57–121 (2007).

[52] Letelier J (2018). A conserved Shh cis-regulatory module highlights a common developmental origin of unpaired and paired fins. Nat. Genet..

[53] Freitas R, Gómez-Skarmeta JL, Rodrigues PN (2014). New frontiers in the evolution of fin development. J. Exp. Zool. Part B Mol. Dev. Evol..

[54] Dahn RD, Davis MC, Pappano WN, Shubin NH (2007). Sonic hedgehog function in chondrichthyan fins and the evolution of appendage patterning. Nature.

[55] Ando H (2005). Lhx2 mediates the activity of Six3 in zebrafish forebrain growth. Dev. Biol..

[56] Ding HL, Clouthier DE, Artinger KB (2013). Redundant roles of PRDM family members in zebrafish craniofacial development. Dev. Dyn..

[57] Neumann CJ, Grandel H, Gaffield W, Schulte-Merker S, Nüsslein-Volhard C (1999). Transient establishment of anteroposterior polarity in the zebrafish pectoral fin bud in the absence of sonic hedgehog activity. Development.

[58] Fischer S, Draper BW, Neumann CJ (2003). The zebrafish fgf24 mutant identifies and additional level of Fgf signaling involved in vertebrate forelimb initiation. Development.

[59] Kucenas S (2008). CNS-derived glia ensheath peripheral nerves and mediate motor root development. Nat. Neurosci..

[60] Smeeton J, Askary A, Crump JG (2017). Building and maintaining joints by exquisite local control of cell fate. Wiley Interdiscip. Rev. Dev. Biol..

[61] Kawakami, K., Asakawa, K., Muto, A. & Wada, H. *Tol2-mediated transgenesis, gene trapping, enhancer trapping, and Gal4-UAS system.**Methods in Cell Biology* vol. 135 (Elsevier Ltd, 2016). 10.1016/bs.mcb.2016.01.011 27443919

[62] Westerfield, M. *The zebrafish book : a guide for the laboratory use of zebrafish (Danio rerio)*. (2000).

[63] Inoue D, Wittbrodt J (2011). One for all-a highly efficient and versatile method for fluorescent immunostaining in fish embryos. PLoS ONE.

[64] Jackman WR, Draper BW, Stock DW (2004). Fgf signaling is required for zebrafish tooth development. Dev. Biol..

[65] Jia, H., Zhu, Y., Xu, K., Pan, G., Liu, X., Qiao, Y., & Wu, F. Efficient cell surface labelling of live zebrafish embryos: wash-free fluorescence imaging for cellular dynamics tracking and nanotoxicity evaluation. *Chem. Sci.* **10**(14), 4062–4068. 10.1039/C8SC04884C (2019). 10.1039/c8sc04884c PMC6461115 31015947

