# Supplementary material for: Developmental independence of median fins from the larval fin fold revises their evolutionary origin
Source: Sci Rep. 2022 May 7;12:7521. doi: 10.1038/s41598-022-11180-1 (PMC9079066; doi:10.1038/s41598-022-11180-1)
Supplement: Supplementary file 1 — Supplementary Information. [file 41598_2022_11180_MOESM1_ESM.pdf]

Title: Developmental independence of median fins from the larval fin fold revises their evolutionary origin.

Authors:
Kazuhide Miyamoto[1], Koichi Kawakami[2,3], Koji Tamura[1], Gembu Abe[1*]

Affiliations:

[1] Department of Ecological Developmental Adaptability Life Sciences, Graduate School of Life Sciences, Tohoku University, Aobayama Aoba-ku, Sendai 980-8578, Japan

[2] Laboratory of Molecular and Developmental Biology, National Institute of Genetics, Mishima, Shizuoka, 411-8540, Japan

[3] Department of Genetics, The Graduate University for Advanced Studies, SOKENDAI, Mishima, Shizuoka, 411-8540, Japan

***Corresponding authors:**

Gembu Abe
Address: Laboratory of Organ Morphogenesis,
Department of Ecological Developmental Adaptability Life Sciences, Graduate School of Life Sciences, Tohoku University,
Aobayama Aoba-ku, Sendai 980-8578, Japan.
Tel: +81-22-795-6677
Fax: +81-22-795-6677
Email: gembu.abe.b5@tohoku.ac.jp

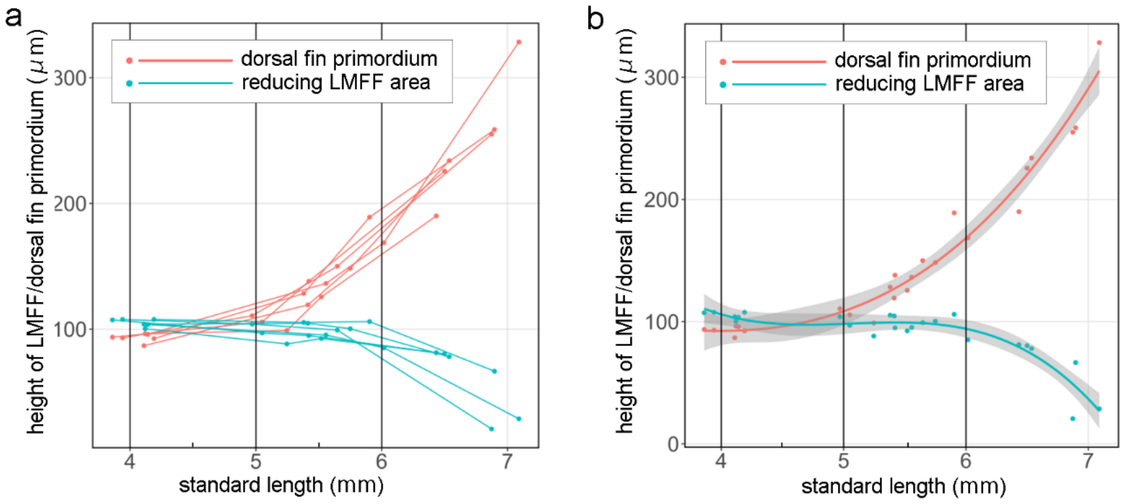

Figure S1. (**a**,**b**) Transition of height of the LMFF/dorsal fin primordium (n = 6). Each line in (**a**) indicates temporal transition of the same individual. (**b**) Local polynomial regression fit of (**a**). The 95% confidence intervals are indicated as grey areas in (**b**).

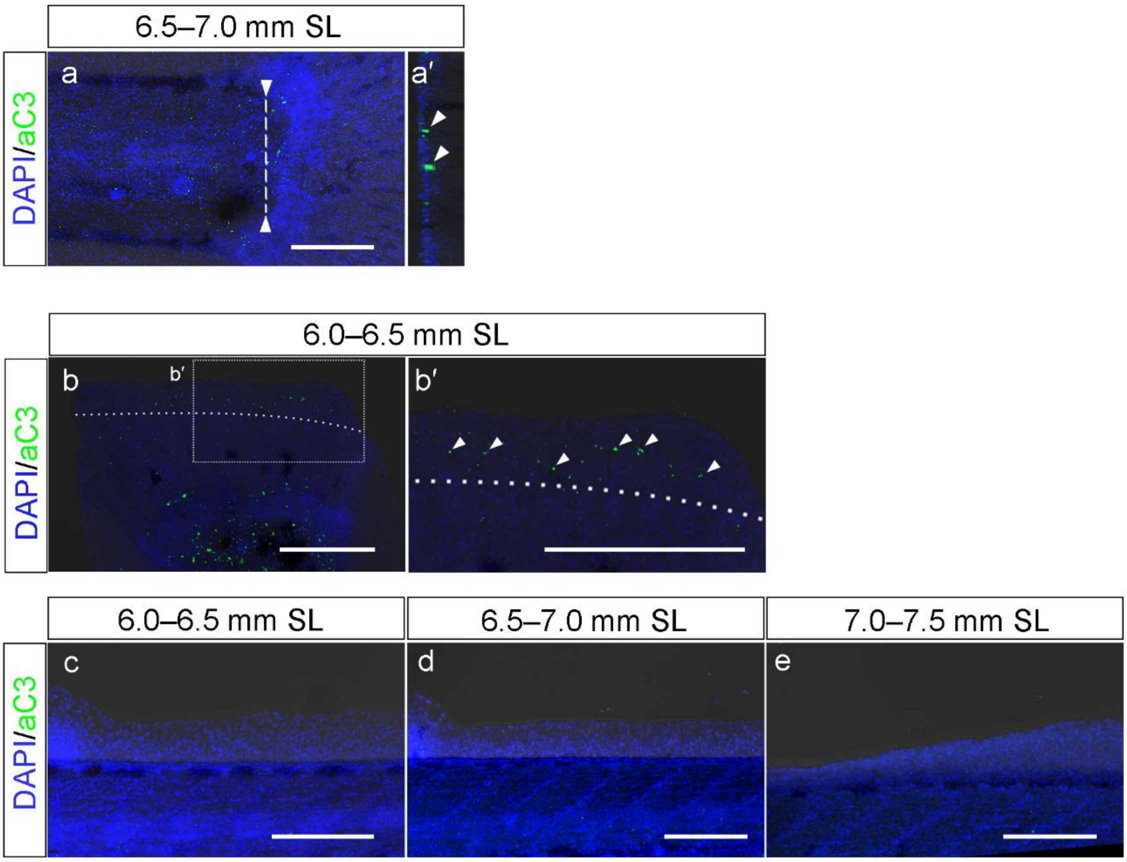

Figure S2. Expression pattern of active caspase 3 in the Juvenile zebrafish. (**a-e'**) Expression pattern of active caspase 3 in proximal part of the developing caudal fin region at 6.5–7.0 mm SL (**a, a'**), the regenerating caudal fin at 6.0–6.5 mm SL (**b, b'**) and the reducing LMFF area in the specimens processed by heating protocol (**c, d, e**). (**a'**) Optical sections proximal part of the developing caudal fin of the dashed line in (**a**). The middle right panel (**b'**) are magnified views of the dashed rectangles in the middle left panel (**b**). Arrowheads in (**a', b'**) indicate examples of apoptotic cell death signals. Scale bars indicate 200 μm.

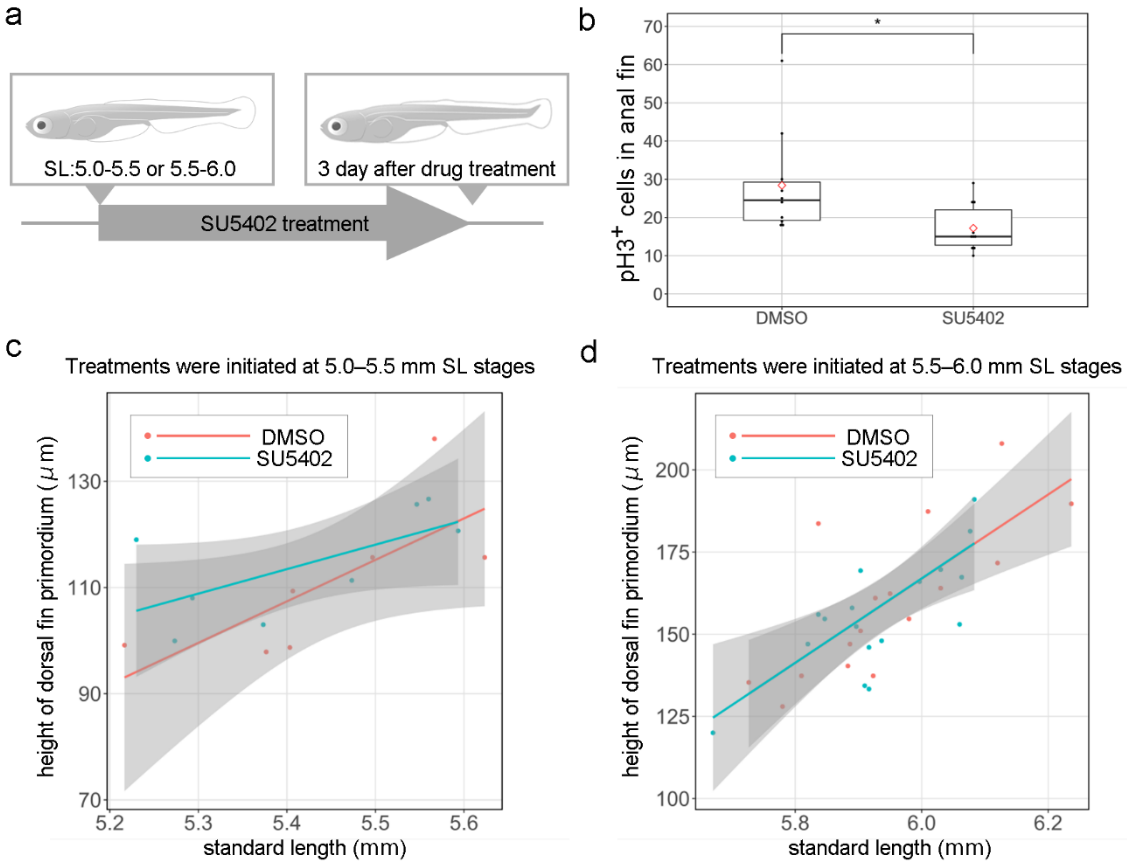

Figure S3. Effect of SU5402 treatment. (**a**) Scheme of SU5402 treatment. (**b**) Boxplots of phospho-histone-H3-positive cells in anal fin primordia after SU5402 treatment that started at 5.0–5.5 mm SL. This proportion was calculated from the number of pH3-positive cells in anal fin primordium. (**c**,**d**) Quantification of dorsal fin primordium height after SU5402 treatments started at 5.0–5.5 mm SL (**c**) and 5.5–6.0 mm SL (**d**). Whiskers in (**b**) show maximum and minimum values within 1.5 times the interquartile range. Boxes show the median and 25th and 75th percentiles. The P value in (**b**) is the result of Welch's t test (P = 0.03447). Standard length/height of the dorsal fin primordium angle slope between trials of DMSO control fish and SU5402-treated fish was not significantly different ((**c**) P = 0.01019; ANCOVA, (**d**) P = 5.931e-06; ANCOVA). The 95% confidence intervals are indicated as grey areas in (**c**,**d**).
